# Machine learning approach for predicting tramp elements in the basic oxygen furnace based on the compiled steel scrap mix

**DOI:** 10.1038/s41598-025-86406-z

**Published:** 2025-01-18

**Authors:** Michael Schäfer, Ulrike Faltings, Björn Glaser

**Affiliations:** 1 Department of Materials Science and Engineering, KTH Royal Institute of Technology, 10044 Stockholm, Sweden; 2 SHS - Stahl-Holding-Saar GmbH & Co. KGaA, Digitalization & AI, 66763 Dillingen, Germany

**Keywords:** Engineering, Materials science, Computer science, Mathematics and computing, Computational science

## Abstract

In the blast furnace and basic oxygen furnace route, pig iron and steel scrap are used as resources for steel production. The scrap content can consist of many different types of scrap varying in origin and composition. This makes it difficult to compile the scrap mix and predict the future chemical analysis in the converter. When compiling the scrap mix, steel manufacturers often rely on experience and trials. In this paper, we present a machine learning approach based on XGBoost to predict the chemical element content in the converter. Data from around 115000 heats were analyzed and a model was developed to better predict the content of the tramp elements copper, chromium, molybdenum, phosphorus, nickel, tin and sulphur at the end of the basic oxygen furnace process. The study shows that it is possible to predict the chemical element content for tramp elements in the converter based solely on data available in advance and routinely collected without the necessity of additional sensors or analysis of input material. Given the nature of scrap classifications for (external) scrap types, this is non-trivial. Furthermore, an online model was implemented, accessible via a defined synchronous interface, which allows to optimize the use of different scrap types by predicting the chemical content at the end of the basic oxygen furnace process and simulating with new combinations of input material. Not all types of steel scrap are always available. With the model developed, new scrap input constellations can now be created to ensure that the quality of the melt is maintained. However, for very accurate predictions, the data from the upstream processes must be of high quality and quantity. Efficient scrap management, monitoring of the scrap input and confusion checks.

## Introduction

The steel industry is currently facing significant challenges due to resource shortages for some materials and high $CO_2$emissions. Steel scrap is a way of countering environmental pollution and energy consumption because, unlike iron ore, it does not require any additional reduction energy to be used in the manufacturing process^1^. Moreover, steel scrap can contain valuable resources such as alloys. The recycling of steel scrap is furthermore economically very attractive, as it is usually cheaper than iron ore-based steel production^2,3^. This is one of the reasons why some steel manufacturers are switching to steel production using electric arc furnace (EAF). However the basic oxygen furnace (BOF) manufacturing process is currently still the most common method of steel production^4^. In the BOF process, pig iron and up to 25% scrap are used. Scrap may contain some chemical elements not desired in steel that cannot be easily removed in the production process, in particular *Cu*, *Ni*, *Co*, *Mo*, *Cr*, *Sn*, *S* and *P*^5–7^. . However, there are elements more noble than iron that cannot be easily removed during the converter or secondary metallurgy process, including *Cu*, *Ni*, *Co*, *Mo*, *Cr* and *Sn*^5,6^. The various chemical elements have a significant influence on the subsequent steel properties such as hardness, strength or toughness^8,9^. Therefore, the scrap used has a considerable influence on product quality and not all scrap types are adequate for the production of certain types of steel. Thus, an accurate prediction of the tramp element amounts in the molten steel based on the charged materials helps to optimize the process and avoid overshooting tramp element targets.

Many steel plants rely on experience for forecasting or use empirical methods to support the control of their processes^10^.This involves regularly taking spectroscopic probes of the charged materials and using physical models to estimate the process outcome. However, the currently available methods are not able to accurately predict future chemical element content^10^. This is mainly due to the complexity of the steel production process and the heterogeneity of some of the charging materials, which makes keeping an accurate datasheet of representative probing results expensive and time consuming. Steel is produced in individual batches using different raw materials, which results in a multidimensional and non-linear process^11^. Generally, machine learning approaches can be helpful in such cases. Published studies on the use of machine learning technologies for the prediction of individual chemical analyses^10,12,13^in steel are limited and focus only on a few selected chemical elements or different chemical elements than in this study, or the prediction of chemical element content of the scrap used in the EAF process from recent EAF process data^14^ instead of the BOF.

The objective of this study is to predict the content of several chemical elements, i.e. *Cu*, *Cr*, *Ni*, *Mo*, *Sn*, *S*, and *P*, in the liquid steel at the end of the BOF process, based on the added charging materials. It should be noted that (external) scrap types are often characterized by size and shape rather than by chemical composition, except for upper boundaries for some tramp elements. Therefore, the prediction of tramp element content in the liquid steel based solely on the scrap type is a non-trivial task. In the BOF route, the elements sulphur and phosphorus are mainly, but not solely, introduced via the pig iron. These elements were included in the analysis as the knowledge of the expected content in the BOF can help to optimize the BOF process. The results can furthermore be transferred to the EAF route, where the introduction of sulphur and phosphorus via additions is relevant. Another important point is the online use of the prediction results in the steel production and planning processes. Therefore, an online model was implemented which can be accessed via a defined synchronous interface.

The dataset (see Section Dataset) used in this study includes 50 different scrap categories. To generate a solid database, an organized scrap yard, scrap sorting and a confusion check are essential. In the steel mill, this is typically done by visual inspections and relies on the operator’s experience. Scrap suppliers often use traditional techniques such as magnetic separation^15^, eddy current separation^16,17^or laser-induced breakdown spectroscopy (LIBS)^18^. In recent years, much progress has been made in the field of machine vision using classical computer vision^19^or machine learning approaches^20–24^ to classify or sort steel scrap. These technologies and systems provide the basis for a database used to control, monitor, and optimize the scrap process in the BOF and EAF, but do not provide information on the chemical content of the scrap or are not applicable for all types of scrap.

This work is organized as follows: Section Methods describes the data set, the algorithm used and the experimental setup. In Section Results and discussion, the results are analyzed and discussed. Finally, in Section Conclusion and future work we summarize our results and make recommendations for future work.

## Methods

**Fig. 1:**
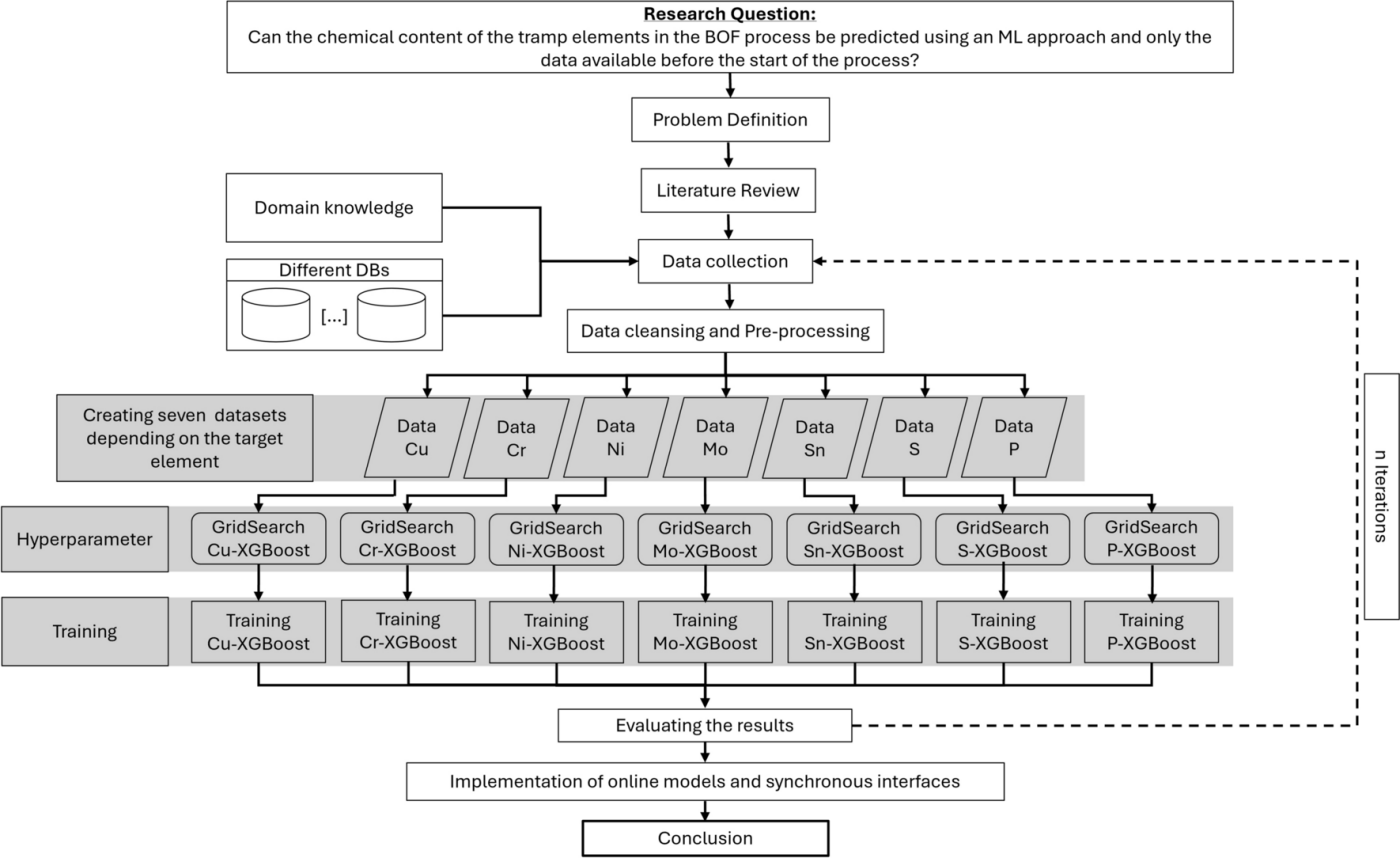
Research framework diagram.

The research framework diagram shown in Fig. 1 presents the research study in a logical and structured way.

To solve the research question, the problem that occurs in the productive environment in industry was first defined. This includes data availability, data quality and which data is available from which point in the process. Another very important point is the subsequent integration or connection into a heterogeneous system landscape. In the next step, possible influencing factors were discussed together with domain experts. These were then extracted from various databases, cleaned and pre-processed. Seven different data sets were then created from this. A grid search was performed on each of the data sets to find the best possible hyperparameters. These were then used to train seven different models. The results of the models were then evaluated. As this is an iterative process, this step was performed several times until the results were deemed satisfactorily and seemed to have reached a plateau. The penultimate step was to implement seven online models and synchronous interfaces, which were then deployed. Finally, a conclusion was created and presented.

**Fig. 2:**
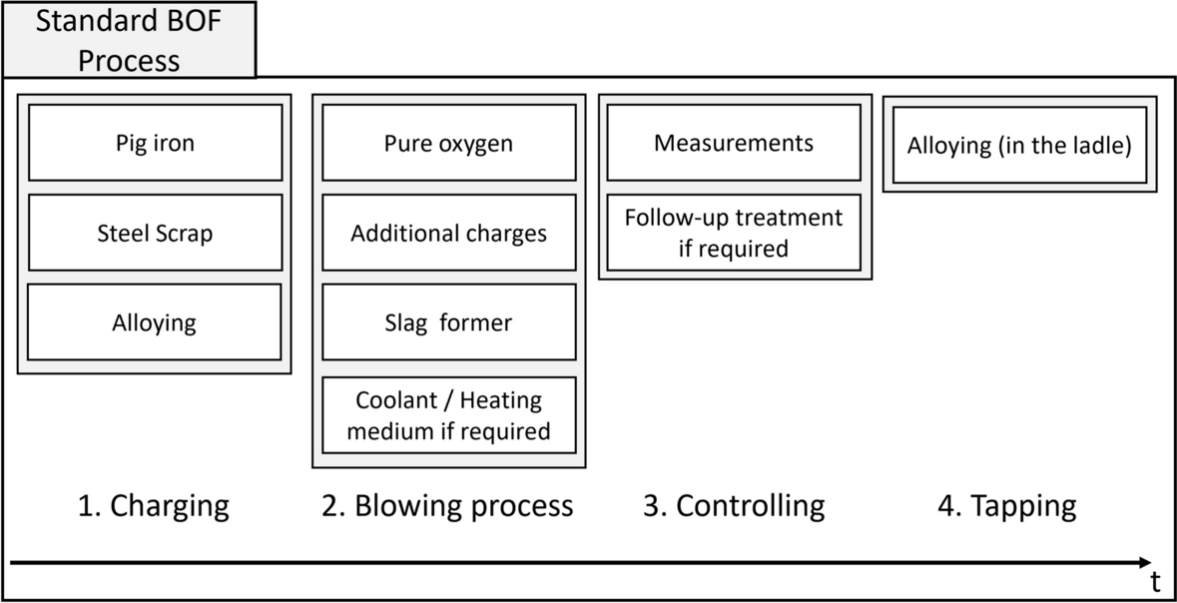
BOF process from filling the materials to tapping.

In this study, we focus on the BOF process for steel making. Figure 2 shows the basic BOF process on which our dataset is based and which defines our prediction targets. In the charging process, various types of steel scrap, pig iron and some alloys are filled into the converter; a converter refers to a large vessel used in the BOF process, where pig iron (from a blast furnace), steel scrap and alloying elements are transformed into steel. Then refining steps are conducted by blowing in pure oxygen and adding additional charges, slag former and cooling or heating mediums as necessary. Next, measurements of chemical elemental contents are taken, and, if required, additional treatment is conducted. At the end of the BOF process, known as tapping, additional alloying elements are added in the ladle.

The chemical elements considered in this study are introduced into the liquid steel from different sources. The pig iron has a carbon content of around 4% to 4, 7% and contains the elements phosphorus, sulphur, silicon and manganese^25,26^, whose tolerable amount depends on the future steel grade. Other trace elements in the molten steel are introduced by the steel scrap, as additives (e.g. coke or ores) and alloying elements (e.g. molybdenum briquettes). The chemical composition of the additives and alloying material is mostly known. However, due to the composition and complexity of the scrap mix used, the exact scrap analysis is either not known or only available for random samples. Therefore, only the weight of the different scrap types added to a particular batch is used in this study and any further scrap analysis or expected contents of trace elements is disregarded. A more detailed description of the dataset used is provided in Section Dataset.

### Dataset

Production data from 2010 to 2024 are used as dataset. Obvious incorrect measurements or incorrect entries were removed from the source database a priori. In this context, data points were classified as obviously incorrect if their value exceeds twice the $\mathrm {99^{th}\, percentile}$ of corresponding datapoints. After eliminating these datapoints, approximately 115000 data records were available.

The converter process is very dynamic and each batch reacts slightly differently. Depending on the dynamics of the steel production process, it can become necessary to make ad-hoc alterations to the initially planned tableau of added materials. In rare cases, this can include the manual input of a material which is not automatically logged in the database systems. This kind of input bears the risk of mistakes and typos when logged a posteriori. Estimating a confidence level for correctness of manually added data is not feasible as there is no monitoring of the data quality for manually added data. Presumably operations personnel usually is diligent in entering correct data, but on the other hand ad-hoc alterations also usually imply unanticipated behavior of the steel batch currently in production and additional stress for the operations personnel, making mistakes more likely. Batches for which manual additions have not been logged in the database cannot be identified and thus remain in the dataset. This is a limitation the authors are aware of. The applied model is however able to handle non-standard-situations as well and the majority of the manual added data entries is expected to be correct.

The used records are data from various heats from the steelworks in Völklingen of Saarstahl AG, Germany. An individual model was trained for each of the target variables, resulting in seven datasets numbered I - VII with identical input features, but different target variable and slightly differing dataset size due to missing target variables measurements. Dataset sizes are provided in Table 1.

**Table 1:** Size of the datasets for each of the respective target elements.

**Dataset**	I	II	III	IV	V	VI	VII
**Target Element**	*Cu*	*Cr*	*Ni*	*Mo*	*Sn*	*S*	*P*
**Size**	115163	115022	115199	115161	115143	115136	115200

Table 2 shows the structure and the number of parameters of the respective datasets.

**Table 2:** Datasets I - VII with same input features and differing target variable.

	**Features**			**Target**						
**Name**	Added material [kg]	Pig iron analysis [$\omega$]	Pig iron weight [kg]	*Cu*	*Cr*	*Ni*	*Mo*	*Sn*	*S*	*P*
**No. of Parameters**	92	22	1	1	1	1	1	1	1	1
**Dataset**	I-VII	I-VII	I-VII	I	II	III	IV	V	VI	VII

The used dataset consists of the following components:

- Feature variables:
  - Chemical analyses of the charged pig iron from the blast furnace (C, Mn, P, W, S, Ni, Mo, Cu, Sn, N, Al, V, Nb, Ti, B, Pb, Te, Bi, As, Ca, Co, Cr) in $\omega$ (mass fraction percentage)
  - Weight of the charged pig iron from the blast furnace in kg
  - Addition quantities of the different types of scrap in kg (Scrap 1, ..., Scrap 50)
  - Addition quantities of the various alloys in kg (Alloy 1, ..., Alloy 7)
  - Addition quantities of other additives in kg (Addition 1, ..., Addition 35)
- Target variables:
  - *Cu*, *Cr*, *Ni*, *Mo*, *Sn*, *S*, *P* chemical element content from probe taken at end of BOF process in $\omega$ (mass fraction percentage)

The “other additives” regarded in the feature variables are for example slag formers, ores, coke and lime. The exact range of used input materials for scrap, alloys and other additives is confidential information, such that they cannot all be provided in detail here. The used scrap consists of internal scrap with corresponding internal scrap type classes as well as external scrap. For external scrap, the used scrap types roughly follow the European scrap type classification, but with some additional more fine-grained internal confidential differentiations. The unit for the pig iron analysis and the target variables is mass fraction percentage. The mass fraction defines the relative proportion of a mass in the total mass of a mixture of substances and is defined as follows^27,28^:

$$\begin{aligned} \omega _{j} = \frac{m_{j}}{\sum m_{i}}, \end{aligned}$$

where $m_{j}$ is the mass of the component *j* and $\sum m_{i}$ is the mass of all mixtures of substances. The mass fraction percentage is then

$$\begin{aligned} \omega _{j} \cdot 100 \% . \end{aligned}$$

When selecting the influencing variables, the data available at the start of the process was intentionally chosen, i.e. charging materials added in the first two process steps (see Figure 2) of the BOF process. Temperature, process times or rinsing intensities were deliberately omitted. These data are only available after tapping and could only be made available as planned data in an online model. The BOF process is very dynamic, such that temperature, process times and rinsing intensity depend on the behavior of the specific batch and are adapted repeatedly during the process rather than being a static value that can be preset in advance. The aim is to see how accurate a prognosis of the chemical element content can be provided based only on the data available at the start of the process.

with identical input features, but different target variable and slightly differing dataset size due to missing target variables measurements. Dataset sizes are provided in Table 1.

shows the structure and the number of parameters of the respective datasets.

**Fig. 3:**
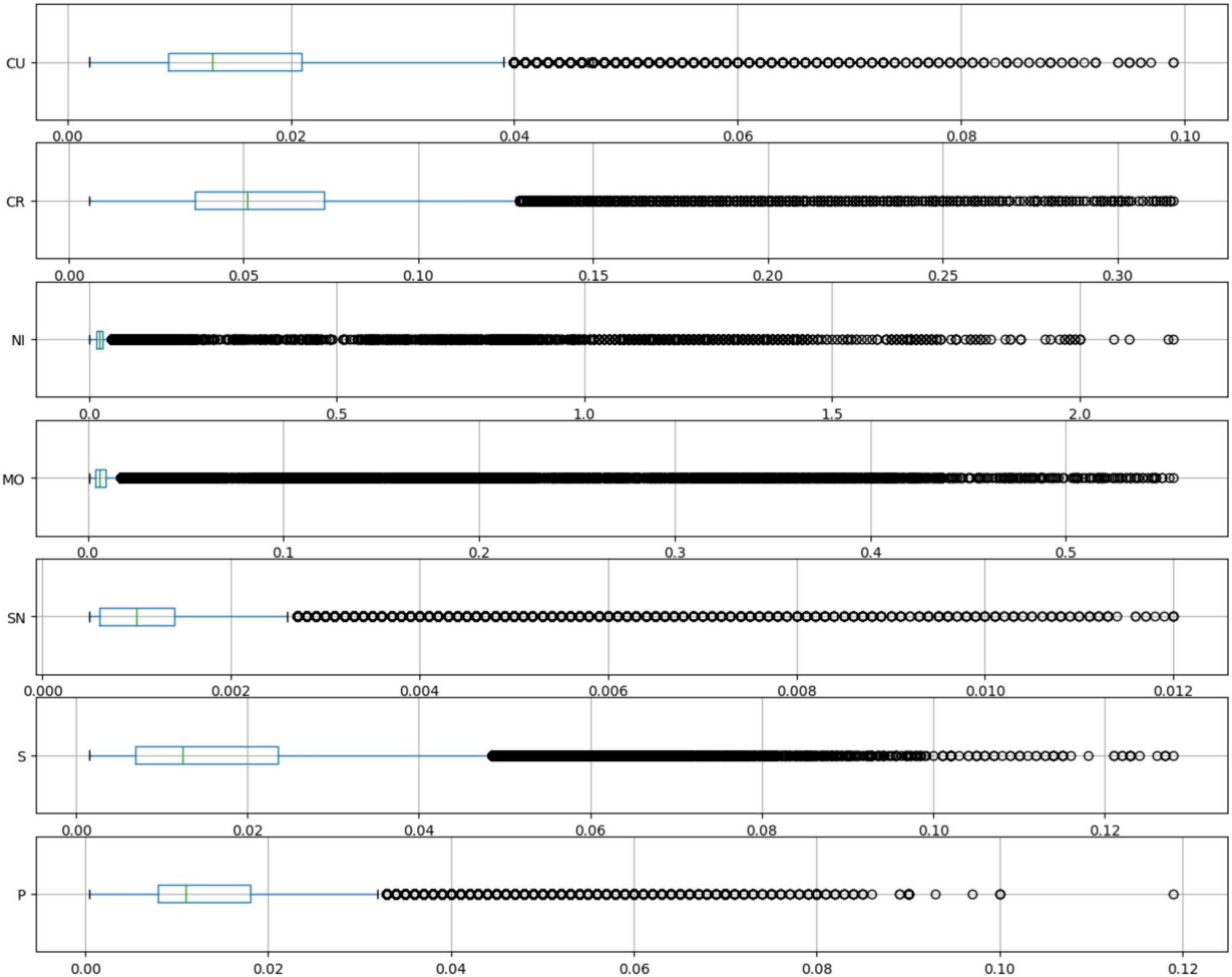
Target variables distributions (in mass fraction %).

Figure 3 shows the distribution of target variables. The box extends from the Q1 to Q3 quartile values of the data, with a vertical line at the median (Q2). The whiskers extend from the edges of box to show the range of the data. By default, the whiskers are limited to $1.5 \cdot IQR, \textrm{with} \; IQR = Q3 - Q1$ from the edges of the box, ending at the farthest data point within that interval. Points outside this range are plotted as separate dots.

### XGBoost

XGBoost^29^(eXtreme Gradient Boosting Decision Tree) is a widely used machine learning algorithm based on a scalable end-to-end tree boosting system which works extremely well on tabular data^30^. XGBoost, a gradient boosted decision tree, is an ensemble learning based technique that uses the results of multiple models to make decisions^31^. In principle, instead of one large holistic model, several simple (so-called weak learner) models are combined, where the successor tree is directly related to the results of the predecessor tree^32^. Basically, each iteration update is based on the result of the previous model. By adding a new tree, an attempt is made to reduce the error between the predicted and the true value. This represents the new model, which is again the basis for the next iteration. The approach is based on the assumption that the combined model improves with each weak model added. The function can be described as follows: For a defined data set ${\mathcal {D}}\{(x_{1},y_{1}),(x_{n},y_{n})\}$, where $x_{i}\in {\mathbb {R}}^m$ = input variables (features) and $y_{i}$ = output (target), the XGBoost model $\phi$ uses *K* decision trees to predict the output $y_i$.

$$\begin{aligned} y_i \approx {\hat{y}}_{i} = \phi (x_{i}) = \sum _{k=1}^{K}{f_{k}(x_{i}),\quad f_{k} \in F } \end{aligned}$$

where $F=\{f(x) = \omega _{q(x)} \}(q : {\mathbb {R}}^{m} \rightarrow T,\; \omega \in {\mathbb {R}}^{T})$ is the space of the different regression trees, defined over their weight vector $\omega$, (also known as CART (Classification and Regression Trees))^29^, *T* the number of leaves in a tree, ${\hat{y}}_{i}$ is the predicted value, and $f_{k}$ the $k^{th}$tree^32^. The loss function, which quantifies the error between the model prediction ${\hat{y}}_{i}$ and the real target value $y_{i}$, is defined as follows:

$$\begin{aligned} {\mathcal {L}}(\phi )=\sum _{i}{l({\hat{y}}_{i}, y_{i})} + \sum _{k}{\Omega (f_{k})}, \end{aligned}$$

where *l* is the distance between *y* and $\hat{y}$ and

$$\begin{aligned} \Omega (f) = \tau T + \frac{1}{2}\lambda \left|\omega \right|^{2}. \end{aligned}$$

$\Omega$ penalizes the complexity of the model. *T* represents the number of leaf nodes and $\omega$ the weights, and $\lambda$ and $\tau$ are the penalty coefficients. The second term $\lambda$ is an additional regularization term and helps to avoid overfitting by penalizing large weight values.

The choice of successive trees is based on a gradient boosting approach, which means the model uses functions as parameters and it is not possible to optimize them using traditional optimization methods in Euclidean space. The model is instead trained in an additive way. Let ${\hat{y}}_i^{(t-1)}= \sum _{k=1}^{t-1}{f_{k}(x_{i})}$ be the prediction of the $i^{th}$ instance $x_i$ at the $(t-1)^{th}$ iteration, then $f_{t}$ is used to minimize the following function:

$$\begin{aligned} {\mathcal {L}}^{t} = \sum _{i=1}^{m}{l(y_{i}, {\hat{y}}_{i}^{(t-1)}+ f_{t}(x_{i})) + \Omega (f_{t})}. \end{aligned}$$

In summary, the $f_{t}$ that improves the model most is added.

### Experimental setup

For each of the target elements *Cu*, *Cr*, *Ni*, *Mo*, *Sn*, *S*, *P*, a separate XGBoost model was trained, using the datasets described in Section Dataset. The respective dataset was split into a train and test set with test set size $= 0.25 \cdot$ train set size, i.e. using $\frac{1}{5}th$ of the dataset for the test set and $\frac{4}{5}th$ for the train set, and a 5-fold cross-validation grid search on the train set was performed to find the best hyperparameters per target element. The used parameter grid for “max_depth” was [4, 5, 6, 8, 10, 15], for “n_estimators” [600, 700, 900] and for “learning_rate” [0.01, 0.015, 0.025], and the root mean squared error was used as evaluation metric. The subsequently selected hyperparameters are provided in Table 3.

**Table 3:** Selected hyperparameters for each of the respective target elements.

**Dataset/Target Element**	I	II	III	IV	V	VI	VII
**max**_**depth**	8	8	4	6	8	8	10
**n**_**estimators**	900	900	900	900	900	900	900
**learning**_**rate**	0.025	0.025	0.015	0.015	0.025	0.025	0.025

The respective model trained with the selected hyperparameters was then evaluated on the test set, calculating the mean average error, mean squared error, root mean squared error and mean average percentage error, and plotting true versus predicted values. Training was conducted on a server with 16 Intel^®^ Xeon^®^ Gold 6342 @ 2.80GHz CPU cores and no GPU; training time per model is provided in Table 4.

**Table 4:** Training times for each of the respective target elements.

**Dataset/Target Element**	I	II	III	IV	V	VI	VII
**Training time (s)**	459.238	454.755	224.878	328.993	425.951	435.782	563.956

The mean average error and mean squared error or root mean squared error are most commonly provided in similar studies. However, without a knowledge of the feature range for a given target element, mean average error, or similarly mean squared error and root mean squared error, as such is not a meaningful measure for the evaluation of the model performance. A low mean average error can be caused by a very good model performance as well as by dominantly small values in the element feature range. We thus provide the mean average percentage error as well.

### Online model

**Fig. 4:**
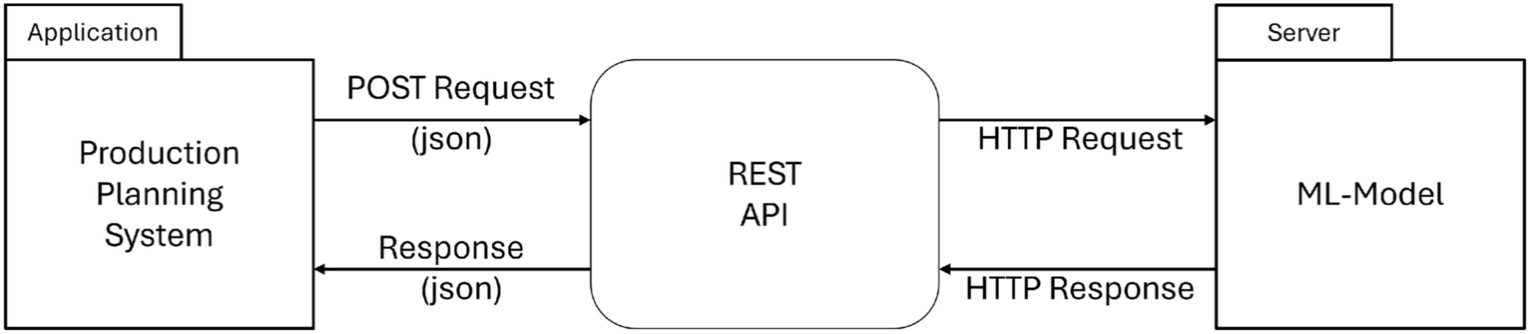
Architecture overview of integration into the production environment using a synchronous REST interface.

For usage in a production setting, the integration of the trained model as an online model is necessary. By providing the machine learning model, the operators and systems that plan the scrap composition are able to optimize the steel scrap mix. A Representational State Transfer (REST)^33^ API was developed so that the trained models can be requested online from a production planning system (PPS). Figure 4 shows the basic architecture of the system. An application such as a PPS requests the API with the required data via a POST request. The respective model is then requested with the data, makes the prediction and returns the model results to the client.

The Listing 1 shows the method for predicting the expected chemical copper amount at the end of the blast furnace process. First, the saved model is loaded once and stored in memory. By loading the model once, subsequent access is significantly faster. In the *predictcu*method, the data is first read in as JavaScript Object Notation^34^ (JSON) from the client and converted into a data frame. This data is then transferred to the loaded model. After the copper model has calculated the predicted value, this is returned as JSON. A total of seven models are loaded and seven different methods for predicting the respective tramp element are offered.
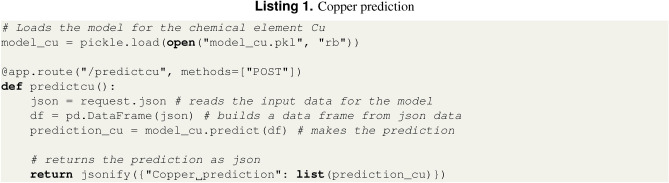


The development and integration of the online model is crucial for several reasons:

1. Real-time predictions and optimization: The model enables a direct prediction of the chemical content in the steel, especially of accompanying elements, i.e. mostly unwanted impurities, using already routinely collected data. In this way, the model can contribute to the optimization of the scrap input in the converter and predict the outcome of the chemical composition of the steel at the end of the BOF process. The ability to operate in near real-time is a key advantage as it allows for ad hoc adjustments rather than relying solely on past data and offline analysis.
2. Efficiency in material handling: Not all types of steel scrap are always available on the market. The developed model helps to simulate and find the best combination of scrap materials to ensure the desired quality of the steel melt. This is especially important as the availability of resources can be unpredictable and flexibility is essential.
3. Cost and resource savings: By predicting results in near real-time, the model helps to improve the quality of the end product and potentially reduce the costs associated with unnecessary testing or over-use of high quality raw materials. It also reduces the need for new sensors.

## Results and discussion

**Fig. 5:**
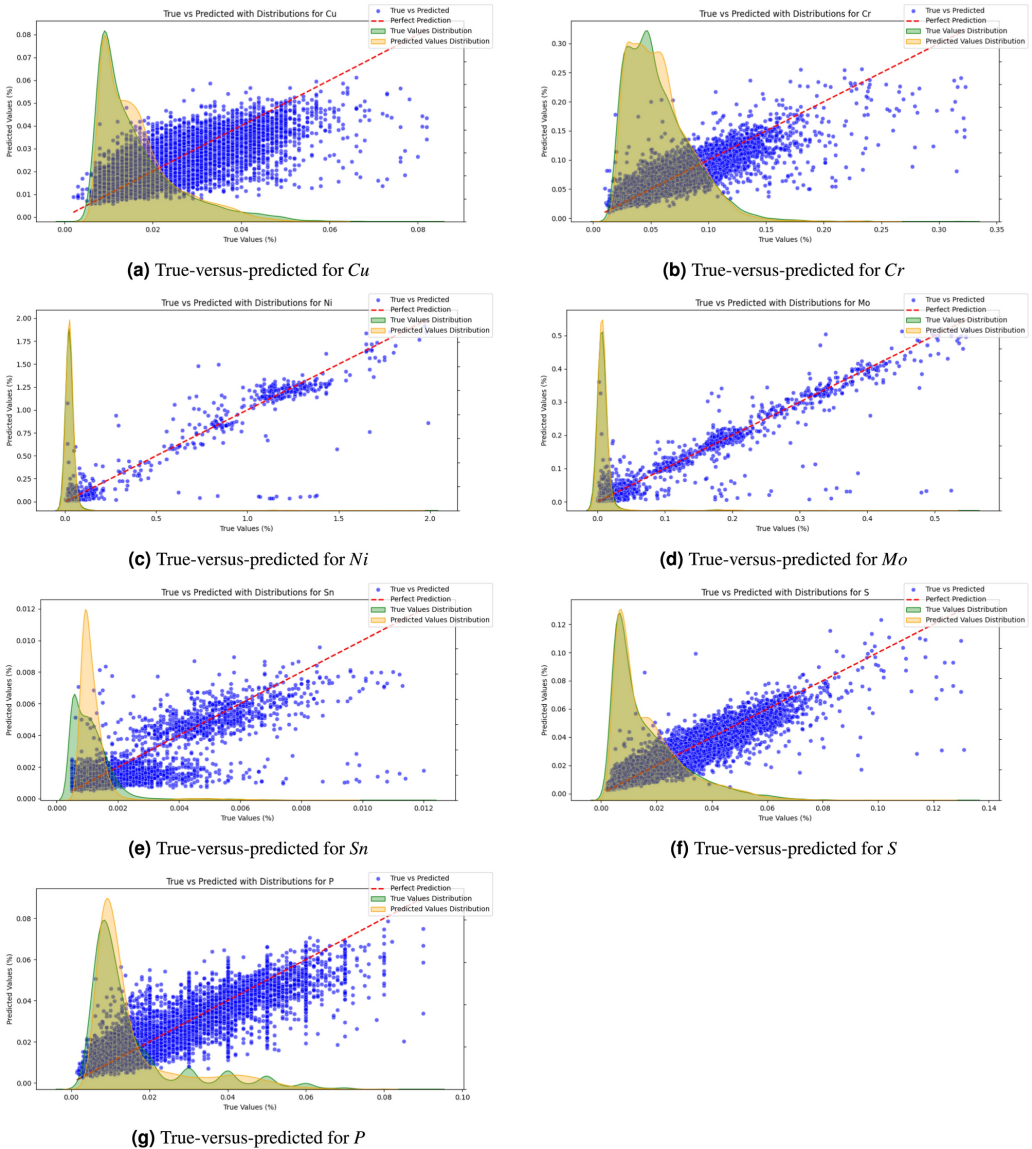
True-versus-predicted value for each target element with kernel density estimate (bandwidth adjust = 1 and “scott” method for bandwidth determination) for true and predicted values: (a) *Cu*, (b) *Cr*, (c) *Ni*, (d) *Mo*, (e) *Sn*, (f) *S*, (g) *P*.

The performance metrics of the trained models are given in Table 5, and the distributions of true-versus-predicted values are shown in Figure 5. The red line in Figure 5 shows an ideal of true value $=$ predicted value and the closer the blue dots signifying individual predictions adhere to this line, the smaller the prediction error is.

**Table 5:** Performance metrics results (MAE - Mean Absolute Error, MSE - Mean Squared Error, RMSE - Root Mean Squared Error, MAPE - Mean Average Percentage Error).

**Dataset**	**Target Element**	**MAE**	**MSE**	**RMSE**	**MAPE**
I	Cu	0.00330	0.00003	0.00551	0.19174
II	Cr	0.00887	0.00023	0.01523	0.15789
III	Ni	0.00839	0.00147	0.03394	0.25112
IV	Mo	0.00316	0.00014	0.01178	0.34847
V	Sn	0.00041	0.00000	0.00060	0.41466
VI	S	0.00316	0.00002	0.00499	0.22148
VII	P	0.00373	0.00003	0.00589	0.26542

Regarding the plotted true-versus-predicted values as well as comparing the performance metrics with the target variables distribution in Fig. 3 and Table 5, a good agreement between true and predicted values is achieved. *Cr* and *Cu* have the highest prediction accuracy in terms of MAPE with the fewest far outliers; in terms of the absolute error metrics MAE, RMSE and MSE, *Sn* has the lowest error, *Cu*, *Mo*, *S*, and *P* lie in a similar middle range and only *Ni* and *Cr* have an MAE above 0.005 percentage points.

Especially for *Ni*, *Mo*, *Sn*, and *P*, and less so for *Cu*, *Cr* and *S*, there is a tendency to predict far too low values in some cases, as visible in Fig. 5. This suggests that some sources for the respective element where either not included in the input parameters for these cases, or respective element content in certain input materials can be well above average in rare cases. E.g. a piece of *Sn*-coated material erroneously ended up in a batch of a scrap type where it is not allowed, leading to a higher *Sn*-amount then what was to be expected in the resultant batch of liquid steel. These comparatively few predictions with higher prediction error are also reflected in the range of MAE vs. RMSE for the respective element. RMSE is more sensitive to large prediction errors then MAE. Thus the closer values for MAE and RMSE are to each other, the less often large prediction errors occur, i.e. as compared to errors in the range of the MAE.

While the absolute error metrics such as MAE, MSE, RMSE give a good overview of the absolute range of prediction errors, allowing an estimate of whether prediction errors are within an acceptable tolerance for a certain elemental amount in a production setting, the MAPE gives a quantitative measure of the model performance as such. Similarly to RMSE, it is however sensitive to individual outliers, especially when the absolute range of values is very low. This is well visible in the correlation between a larger difference between MAE and RMSE for e.g. *Ni*, *Mo*, *Sn*, and *P*, and higher MAPE values for these elements.

*Ni* is the element where MAE, MSE, and RMSE are all highest, followed by *Mo* and *Cr*. For *Cr* however, the low MAPE shows that this is rather due to an overall higher range of element amount values than to a subpar model performance. The element *Sn* on the other hand with the highest MAPE has the lowest absolute error metrics MAE, RMSE, MSE, showcasing that in this case the relative error may be comparatively high, but the model performance is still very good in terms of usefulness for a steel production setting.

As can be seen in the box plots in Fig. 3, the distribution of the target variables for all seven elements, but in particular for *Ni* and *Mo*, has a comparatively compact range in which the majority of data points lies and a few batches with significantly higher elemental amounts forming the dotted outliers in the box plots.

In the true-vs-predicted-plots in Fig. 5, this is not as well visible because the very large bulk of low values form overlapping dots in the plots such that the proportions of number of low values compared to higher elemental amounts is not as discernible anymore. In particular, *P*, *Sn* and *Cu* have comparatively few outlier values, i.e. dots above the whiskers in the box-plots in Fig. 3, which could be another explanation for the models tendency to predict slightly too low values for higher true values for these elements. As visible in Fig. 5, the bulk of dots slope below the red diagonal for higher true values. Nevertheless, even for high true values there is still a clear diagonal upward trend in the plotted dots. For *Cr*, *Ni*, *Mo*, and *S* with more high outlier values in the target variable, forming an almost solid line of dots above the whiskers in the box-plots in Fig. 3, this trend is even stronger, showing the ability of the used XGBoost models to perform well even on such unbalanced challenging data.

If additional process data from during the BOF process were included in the used dataset, effects such as the losses during oxygen blowing would probably be influencing the models in a more fine-grained manner. However, by learning statistical correlations between the used materials and the chemical probe results at the end of the BOF process, an implicit, albeit more coarse, understanding of the effects is learned by the models used. As explained in Section Dataset, the data used in this study intentionally consisted only of parameters known before the start of the converter phase, omitting process data not available until during the BOF process.

Our study also has some limitations which stem from the used process data. In the past, amounts for some alloying materials, especially for *Ni* and *Mo*, had to be entered into the database system manually. It is possible that in some cases, this could have resulted in wrong data entries. Detecting that kind of error in retrospective for a particular batch is however very difficult, and limiting the data to the nearer past when this error was less likely would have decreased the overall amount of data too severely. Thus the actual prediction accuracy of the models, especially for *Ni* and *Mo*, might be higher than reported in this study. Another limitation is that some of the found hyperparameters were always at the end of the chosen grid. Experimenting with a larger range in the grid search was tried, but had to be aborted due to lack of computational resources.

## Conclusion and future work

The aim of this study was not to show that XGBoost outperforms other models. Rather, XGBoost was chosen because it is a well established standard model for the type of tabular data used in this study. What the study shows is that it is possible to predict the content of tramp elements at the end of the BOF process based solely on the scrap type (without the chemical composition of this scrap type being known) resp. other charging material (without its chemical composition known) and the chemical composition of the pig iron. Given the specification for (external) scrap types, the finding that tramp element content in the liquid steel can be predicted based solely on the scrap type is a non-trivial result. State-of-the-art for the BOF process is to take probes of the different input materials at certain intervals and have datasheets with resulting estimated chemical compositions. Depending on the homogeneity of the material and variance on composition over time, these estimates will be more or less accurate. At the same time this is expensive and time consuming. These estimates are then used in a physical model of the BOF to predict tramp element content in the liquid steel at the end of the BOF process. Based on this physical model, a scrap (and other input material) composition for the to-be-produced batch is chosen. The presented approach shows that the regular manual updating of the datasheets as well as the physical model for predicting element content at the end of the BOF process based on these datasheets could both be replaced by an automated data-driven system using well-established ML models, not requiring additional sensors or data collection apart from what is already routinely collected.

In this work, seven machine learning models based on XGBoost have been trained to predict the chemical element content of tramp elements in the first phase of the steelmaking process. Data from approx. 115000 heats were used to train and test an XGBoost machine learning algorithm. In addition, a k-fold cross-validation grid search was performed on the training set to find the best hyperparameters per target element. It should be emphasized that these results were achieved using only the process data that is persisted as default in the steelworks. The results of the study show that machine learning models can predict outcomes in very complex processes such as the BOF process. When comparing our results with results published in the literature for the EAF route, such as^14^, results are in a comparable accuracy range despite the BOF process being chemically more complex than the EAF process. Table 6gives an overview of the results published in^14^for four EAFs and the results for the BOF presented in this study. In particular, it should be noted that the authors in^14^ also used process parameters such as process times, temperatures, energy and oxygen consumption which we purposefully omitted.

**Table 6:** Performance metrics results RMSE and mean of target element data distribution (Ovako steel, Fundia special bar, Sandvik materials technology and Fundia armering from^14^ for EAF, and Völklingen steelworks from this study for BOF)).

**Target Element**	**Ovako steel**		**Fundia special bar**		**Sandvik**		**Fundia armering**		**Völklingen**	
	**RMSE**	**Mean**	**RMSE**	**Mean**	**RMSE**	**Mean**	**RMSE**	**Mean**	**RMSE**	**Mean**
Cu	0.029	0.187	0.03	0.24	0.03	0.2	0.041	0.359	0.00551	0.01674
Cr	0.08	0.28	0.02	0.07	0.71	15.74	0.027	0.118	0.01523	0.05846
Ni	0.06	0.16	0.02	0.13	0.55	7.42	0.025	0.168	0.03394	0.04103
Mo	0.02	0.04	0.01	0.03	0.1	1.05	0.005	0.026	0.01178	0.01442
Sn	0.002	0.009	0.003	0.017	-	-	0.005	0.017	0.00060	0.00125
S	0.004	0.025	0.006	0.037	0.003	0.013	0.005	0.043	0.00499	0.01740
P	0.003	0.008	0.003	0.011	0.002	0.021	0.005	0.018	0.00589	0.01614

Steel production via the BOF route is a multidimensional process and not all data influencing the metallurgical process is available. To improve the results even further, much more process data would have to be collected, analyzed and made available to the model as input data.

This work is a step towards using machine learning to compile the steel scrap more targeted for a particular batch in order to achieve an optimal chemical analysis in the converter with the most cost-efficient input materials. Topics for future studies could be for example:

- It is notable that in the present experiments, the largest number of estimators was always chosen in the grid search. It should be evaluated how larger estimator numbers affect model results.
- A comparison with other tree-based machine learning algorithms such as CatBoost^35^ or a deep learning approach would be very interesting.
- The transfer of the models to the EAF process is worth investigating.
- At present, the XGBoost models used in the online model are regularly manually retrained, however, in the future, the presented architecture for the online model could include a regular automated retraining.
- Further studies should investigate the possibility of attributing predicted chemical element amount in the converter to the individual input materials. This would allow to calculate the approximate optimal quantity of input materials in advance instead of finding it via a simulation with different scrap mixes or through a physical model.

## Data Availability

The data that support the findings of this study are available from Saarstahl AG but restrictions apply to the availability of these data, which were used under license for the current study, and so are not publicly available. Data are however available from the authors upon reasonable request and with permission of Saarstahl AG.
